# Adipose Inflammation Initiates Recruitment of Leukocytes to Mouse Femoral Artery: Role of Adipo-Vascular Axis in Chronic Inflammation

**DOI:** 10.1371/journal.pone.0019871

**Published:** 2011-05-20

**Authors:** Sumihiko Hagita, Mizuko Osaka, Kentaro Shimokado, Masayuki Yoshida

**Affiliations:** 1 Life Science and Bioethics Research Center, Tokyo Medical and Dental University, Bunkyo-ku, Tokyo, Japan; 2 Department of Vascular Medicine, Tokyo Medical and Dental University, Bunkyo-ku, Tokyo, Japan; 3 Research Fellow of Japan Society for the Promotion of Science, Chiyoda-ku, Tokyo, Japan; Pennington Biomedical Research Center, United States of America

## Abstract

**Background:**

Although inflammation within adipose tissues is known to play a role in metabolic syndrome, the causative connection between inflamed adipose tissue and atherosclerosis is not fully understood. In the present study, we examined the direct effects of adipose tissue on macro-vascular inflammation using intravital microscopic analysis of the femoral artery after adipose tissue transplantation.

**Methods and Results:**

We obtained subcutaneous (SQ) and visceral (VIS) adipose tissues from C57BL/6 mice fed normal chow (NC) or a high fat diet (HF), then transplanted the tissues into the perivascular area of the femoral artery of recipient C57/BL6 mice. Quantitative intravital microscopic analysis revealed an increase in adherent leukocytes after adipose tissue transplantation, with VIS found to induce significantly more leukocyte accumulation as compared to SQ. Moreover, adipose tissues from HF fed mice showed significantly more adhesion to the femoral artery. Simultaneous flow cytometry demonstrated upregulation of CD11b on peripheral granulocyte and monocytes after adipose tissue transplantation. We also observed dominant expressions of the inflammatory cytokine IL-6, and chemokines MCP-1 and MIP-1β in the stromal vascular fraction (SVF) of these adipose tissues as well as sera of recipient mice after transplantation. Finally, massive accumulations of pro-inflammatory and dendritic cells were detected in mice with VIS transplantation as compared to SQ, as well as in HF mice as compared to those fed NC.

**Conclusion:**

Our *in vivo* findings indicate that adipose tissue stimulates leukocyte accumulation in the femoral artery. The underlying mechanisms involve upregulation of CD11b in leukocytes, induction of cytokines and chemokines, and accumulation of pro-inflammatory cells in the SVF.

## Introduction

Abdominal obesity has been shown to be strongly related to systemic inflammatory state, including the development of vascular diseases and metabolic complications such as dyslipidemia, hypertension, and diabetes mellitus. Recent studies have provided ample evidence to support the importance of low-grade but sustained inflammation in this process. Adipose tissue produces a wide variety of pro-inflammatory cytokines and chemokines, including IL-6 and monocyte chemoattractant protein-1 (MCP-1). These locally produced cytokines recruit immune cells such as monocytes/macrophages, lymphocytes, and dendritic cells (DCs) toward adipose tissues, which aggravate systemic inflammation. Simultaneously, macrophages recruited to adipose tissues then produce pro-inflammatory cytokines or chemokines to further develop and sustain the inflammatory status. This inflammatory cascade in turn may advance atherosclerosis in the large artery.

As previously reported, the phenotypic variety of macrophages is quite diverse, and dependent upon the properties of inflammation and activation *in situ* [1]–[3]. In the stromal vascular fraction (SVF) of adipose tissues, macrophages are generally classified into M1 (F4/80^+^/CD11c^+^) or M2 (F4/80^+^/CD11c^−^) state [4]–[7]. Classically activated M1 macrophages, induced by proinflammatory mediators such as lipopolysaccharide, secrete high levels of proinflammatory cytokines (TNFα, IL-6, IL-12) [8]–[10]. In contrast, alternatively activated M2 macrophages induced by exposure to IL-4 and IL-13 secrete high levels of anti-inflammatory cytokines [11]–[13]. Therefore, the balance between these 2 types of macrophages can regulate the inflammatory status of adipose tissues. In a more recent study, another inflammatory M1 macrophage with a high level of CD11b was identified in the SVF of adipose tissues obtained from individuals who consumed a high fat diet (HF), which correlates with the inflammatory status seen in obese individuals [14]–[15].

Given the close connection between adipose tissue and inflammation, it is critical to assess the role of adipose inflammation in vascular dysfunctions such as atherosclerosis. However, it is not known whether a direct link between inflammation in adipose tissue and that in vasculature is present in the context of atherosclerosis. In the present study, we used a real-time imaging device to visualize vascular inflammation in mice and were able to document that inflammatory adipose tissue directly induces vascular inflammation, as manifested by leukocyte recruitment to the femoral artery. Our *in vivo* findings provide critically important evidence of a mechanistic link between obesity and atherosclerosis.

## Materials and Methods

### Animals

Male C57BL/6J mice (7 weeks of age; day 0) were obtained from Oriental Yeast (Tokyo, Japan), and fed with normal chow (NC) (Clea Japan, Inc., Japan) or a high fat diet (HF) (20% tallow, 1.25% cholesterol, Clea Japan, Inc., Japan). Both food and water were provided ad libitum. The all animal experiments were approved by the ethical committee for animal experimentation of Tokyo Medical and Dental University, Tokyo and conducted according to the institutional guidelines.

### Adipose tissue transplantation

We transplanted 0.05 g of subcutaneous (epidermal adipose tissue, SQ) or visceral (epididymal adipose tissue, VIS) adipose tissue and skeletal muscle (anterior compartment of the hind limb muscle) harvested from donor mice (day 0, 7 weeks of age) into the perivascular area of the right femoral artery of recipient mice (day 0, 7 weeks of age). In the experiments using HF-feeding, adipose tissue was taken from mice under HF or NC for 18 weeks and transplanted to the recipient mice at 7 weeks of age (HF 18w, NC 18w).

### Intravital microscopy

Intravital microscopic (IVM) examination of the contralateral femoral arteries was performed 1 week after adipose tissue transplantation, as described previously [16]–[17]. In brief, mice were anesthetized with pentobarbital and mechanically ventilated so as to maintain a normal acid-base balance. Rectal temperature was kept at 36.0–37.0°C with a heating pad and infrared heat lamp. After injection of rhodamine 6G chloride [Molecular Probe; 0.3 mg/kg in 300 µl of PBS (−)] into the right femoral vein, the left femoral artery was visualized using a fluorescent microscope (BX51WI, Olympus, Tokyo) equipped with a water immersion objective (×20). Epifluorescence was illuminated by a 100 W fluorescent lamp source and images were directly captured to a PC via a CCD camera (CoolSnap HQ, Olympus, Tokyo, Japan). Each experimental group consisted of at least 8 mice. Serum samples and injured femoral arteries were obtained and immediately frozen, then stored at −20°C until the time of study. In some experiments, a rat anti-mouse CD11b antibody (M1/70, Southern Biotechnology) and isotype-matched control IgG (rat IgG2b, κ, Biolegend) were intravenously injected (50 µg/mouse) at 2 hours before IVM analysis.

### Image analysis

Leukocyte adhesion was clearly visualized on the anterior half of the vessel portion facing the objective. All images were analyzed using an image analysis program (Meta morph) in accordance with the manufacturer's protocol, as previously described [16]–[17]. In brief, the number of adherent cells (i.e., those that did not move for 3 sec during the 1 minute recording period) was counted along the region of interest (ROI), a 100×100-µm rectangle segment of the vessel, and expressed as the number of adherent cells/10^4^/µm^2^ of the vessel surface.

### Quantification of inflammatory cytokines and chemokines by ELISA

To detect the levels of inflammatory cytokines and chemokines in serum of recipient mice transplanted adipose tissue of mice at 7 weeks of age (day 0) or fed HF diet for 18 weeks (HF 18w) and adipose tissue removed from donor mice at day 0 or fed HF diet for 18 weeks (HF18w), we conducted ELISA assays. Anti-mouse IL-6 (Endogen), and MCP-1 (R&D) and MIP-1β (R&D) monoclonal antibodies in PBS were adsorbed in microtiter-plate wells overnight at room temperature. Plates were washed with 0.02% Tween 20 in PBS and blocked with 5% normal goat serum in PBS for 1 hour at room temperature, then washed 3 times with 0.02% Tween 20 in PBS. Samples of murine serum and lysates from subcutaneous or visceral adipose tissue were added to the wells, and incubated for 1 hour at room temperature. After washing the plates 3 times, biotinylated anti-mouse IL-6 (Endogen), MCP-1 (R&D), and MIP-1β (Life Span Biosciences) monoclonal antibodies were diluted with PBS, and added to each well. After 1 hour of incubation at room temperature, horseradish peroxidase-streptavidin was added and the plates were incubated at room temperature for 30 minutes. After another wash, immunoreactive protein was developed by addition of tetramethylbenzidine peroxidase substrate. After 30 minutes, the reaction was stopped by addition of 0.6 N H_2_SO_4_, and absorbance was measured at 450 nm. A sample concentration was also obtained for comparison with use of a standard curve.

### Flow cytometric analysis of circulating leukocytes

To detect the expression intensity of cell surface CD11b and intracellular oxidative stress, white blood cells were prepared from 2 recipient mice per each condition (sham, SQ, and VIS) by hemolyzing whole blood. The cells were incubated with anti-mouse CD11b (Serotec, USA) for 45 minutes on ice, followed by an FITC-conjugated secondary antibody (R&D Systems, Inc., USA) for 45 minutes on ice, and finally incubated with dihydroethidium (DHE) (1∶250) for 25 minutes. After 3 washings, fluorescence activity was detected from cell fractions containing 5000 cells using a FACS caliber at 480 nm (CD11b) or 580 nm (DHE), and the data were analyzed with CellQuest software (Becton Dickinson). When fluorescence activity was detected, data was obtained for granulocytes and monocytes using gating definitions as previously reported [18]–[19].

### Flow cytometric analysis of cell population in stromal vascular fraction of adipose tissue

The stromal vascular fraction (SVF) of adipose tissue was isolated from SQ and VIS adiose tissue of donor mouse at 7 weeks of age (day 0) or fed HF diet for 18 weeks (HF 18w). The obtained adipose tissue was minced and incubated in PBS with heparin (5 U/ml) for 30 seconds to remove circulating blood cells. Next, the suspension was centrifuged at 1000× *g* for 8 minutes and collected adipose tissue was incubated with type 2 collagenase in Tyrode's buffer (137 mM NaCl, 5.4 mM KCl, 1.8 mM CaCl_2_, 0.5 mM MgCl_2_, 0.33 mM NaH_2_PO_4_, 5 mM HEPES, 5 mM glucose). The digested adipose tissue solution was centrifuged at 1000× *g* for 8 minutes, and pellets containing SVF were resuspended in PBS and filtered through a 36-µm nylon mesh, then washed twice. Isolated cells were incubated with anti-mouse antibodies (FITC-F4/80, PE-CD11c, Alexa488-CCR7, Bio Legend; CD11b, Alexa647-CD86, Alexa647-CD204, Serotec) for 45 minutes on ice. After 3 washings, fluorescence activity was detected from 5000 cell fractions using a FACS caliber and the data were analyzed with CellQuest software (Becton Dickinson). When fluorescence activity was detected, data were obtained from monocyte/macrophage and dendritic cells (DCs) subsets using gating definitions as previously reported [14], [18]–[20]. The cell population in SVF consisted of M1 (F4/80^+^/CD11c^+^) and M2 (F4/80^+^/CD204^+^) macrophages, activated monocytes (CD11b^+^/CD11c^+^), and 2 subpopulations of mature dendritic cells (DCs) (CD11c^+^/CCR7^+^, CD11c^+^/CD86^+^), as previously reported [5], [8], [14], [21]–[27].

### Statistical analysis

Data are expressed as the mean value ± SEM. One-way ANOVA with a Tukey post-hoc test or two-tailed unpaired t test was used to estimate statistical significance at p<0.05.

## Results

### Transplantation of adipose tissue induced leukocyte adhesion to mouse femoral artery

**Figure 1:**
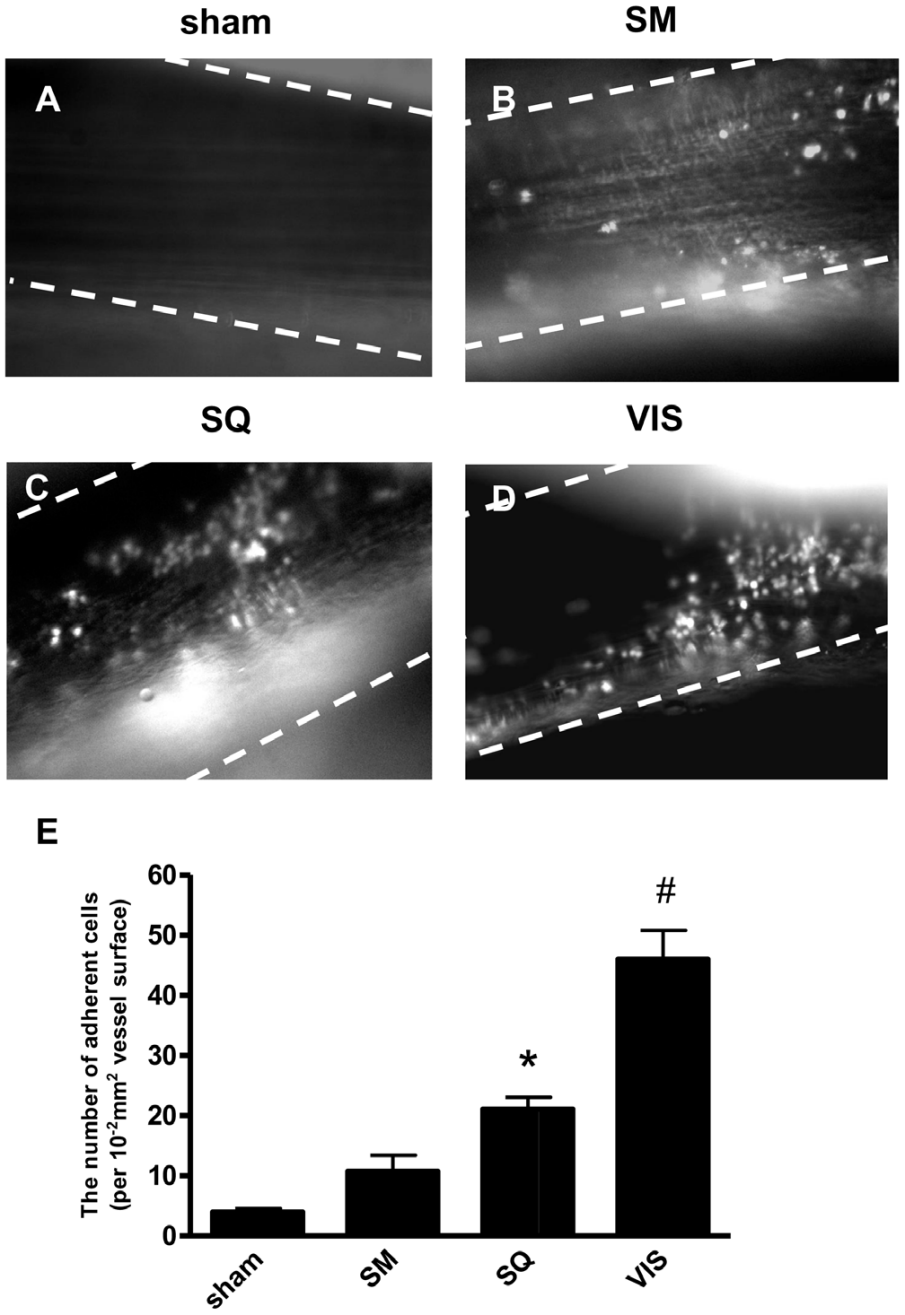
Leukocyte adhesive interactions in arteries of mice with transplanted adipose tissue. Shown are representative images from **A:** sham operated mouse, **B:** skeletal muscle muscle (SM) transplanted mouse, **C:** subcutaneous adipose tissue (SQ) transplanted mouse, and **D:** visceral adipose tissue (VIS) transplanted mouse. **E:** The numbers of adherent cells were quantitated as described in Methods. Values are shown as the mean ± SEM of 10 mice in each group. *P<0.01 vs. sham operation group, ^#^P<0.01 vs. SQ group.

Seven days after transplantation of either SQ or VIS adipose tissue, we observed the left femoral artery of recipient mice and noted that the number of adherent leukocytes was significantly increased as compared with mice that underwent a sham operation (Sham) or those with skeletal muscle transplantation (SM) (**Figure 1A, B**). Between the 2 types of transplanted adipose tissues, VIS induced more prominent leukocyte recruitment than that observed with SQ transplantation (**Figure 1C, D**). Transplantation of both types but not sham operation enhanced leukocyte recruitment in a time-dependent manner (**Figure S1**).

### HF accelerated inflammatory property of mouse adipose tissue

**Figure 2:**
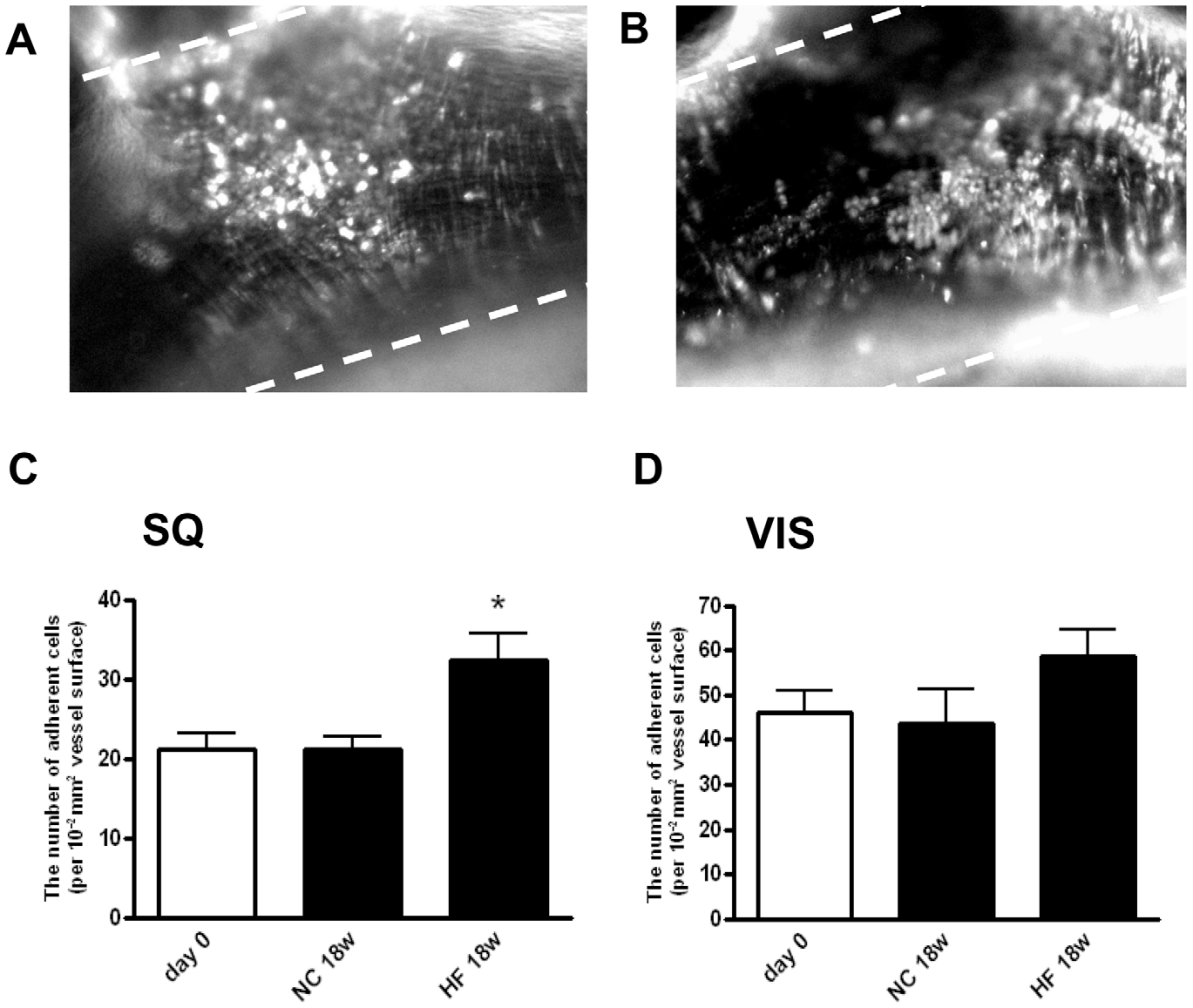
Leukocyte adhesive interactions in arteries of mice with transplanted adipose tissue from donor mice fed a high fat diet. Shown are representative images from mice with **A:** transplanted subcutaneous adipose tissue (HF SQ), **B:** transplanted visceral adipose tissue (HF VIS). **C, D:** The numbers of adherent cells in the vasculature of animals at day 0 and fed NC or HF for 18 weeks from day 0 were quantified as described in Methods. Values are shown as the mean ± SEM of 10 mice in each group. *P<0.05 vs. day 0 and NC groups.

We then examined the effect of an HF on adipose tissue-induced vascular inflammation. Mice were fed an HF or NC for 18 weeks (HF18w or NC18w). The HF feeding for 18 weeks significantly increased body weight as compared to NC (**Figure S2**). Adipose tissues (VIS or SQ) were taken from these mice and transplanted into recipient mice. IVM analysis of the recipient mice was carried out 7 days after transplantation. As shown in **Figure 2**, SQ prepared from HF18w significantly enhanced leukocyte recruitment when compared to those from NC 18w. Interestingly, VIS prepared from HF18w increased leukocyte recruitment, though not statistically significant.

### Levels of inflammatory chemokines and cytokines in donor adipose tissue, and recipient serum

**Figure 3:**
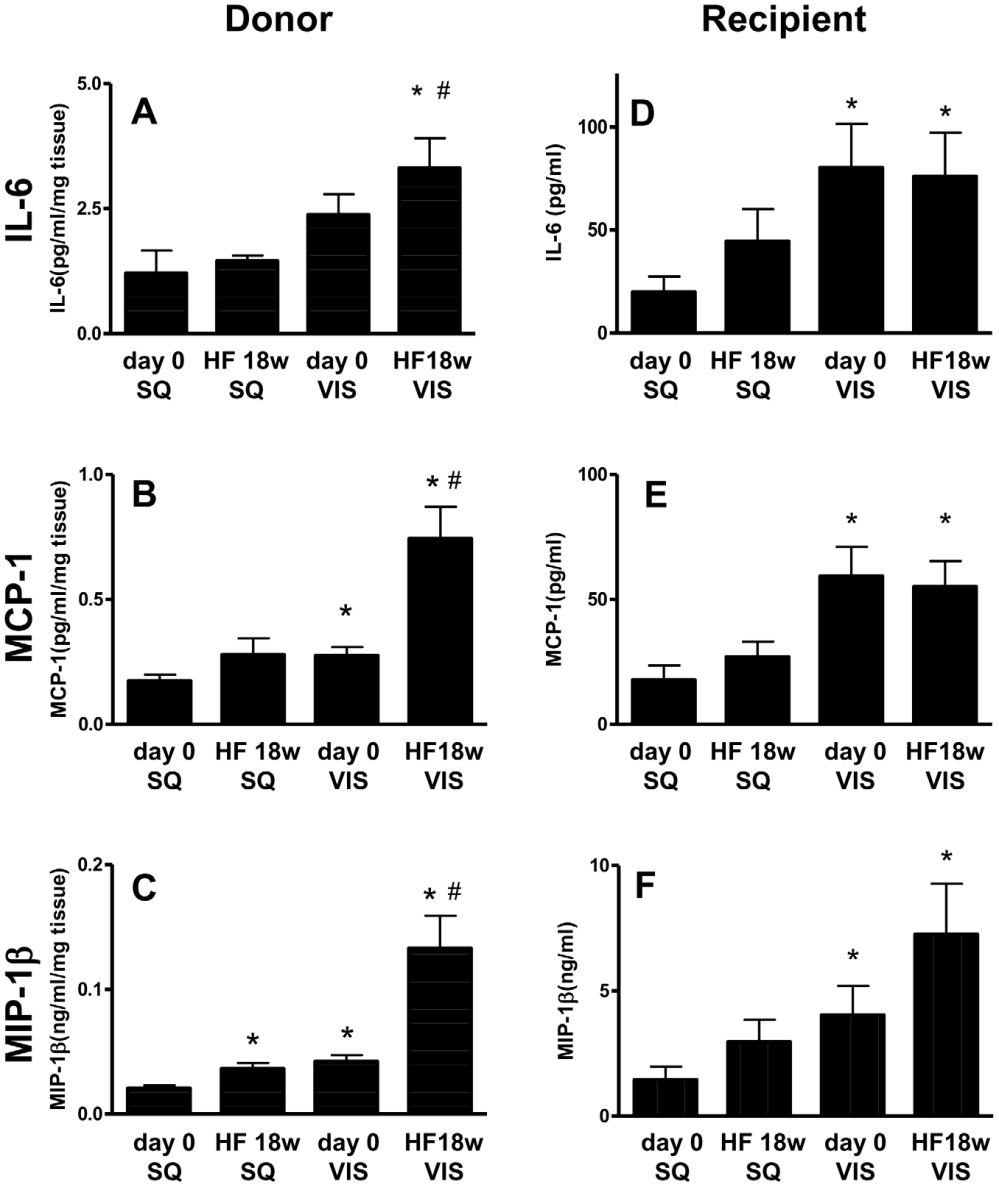
Cytokine and chemokine levels in subcutaneous (SQ) and visceral (VIS) adipose tissue of donor mice at 7 weeks of age (day 0) and fed a high fat diet for 18 weeks (HF), and recipient serum samples. **A, D:** IL-6, **B, E:** MCP-1, **C, F:** MIP-1β Those levels were quantified by ELISA. Values are shown as the mean ± SEM of 6 mice in each group. *P<0.05 vs day 0 SQ group.

To elucidate the molecular mechanisms underlying adipose tissue-induced inflammatory response, cytokine levels were measured using adipose tissue lysates prepared from mice at day 0 and HF18w. The levels of MCP-1 and IL-6 were significantly elevated in VIS adipose tissue from HF18w (**Figure 3A, B**), that were not changed in SQ from HF18w. The level of MIP-1β was significantly elevated in both SQ and VIS from HF18w (**Figure 3C**). We then examined whether these inflammatory profiles in donor adipose tissues reflected vascular inflammation present in the recipient mice. As shown in **Figure 3D, E, F**, VIS transplantation increased the serum levels of MCP-1, IL-6, and MIP-1β in the recipient mice which was not observed in SQ transplantation. Furthermore, VIS-triggered MIP-1β induction was significantly enhanced in HF18w.

### Integrin expression and oxidative stress in granulocytes and monocytes after adipose tissue transplantation

**Figure 4:**
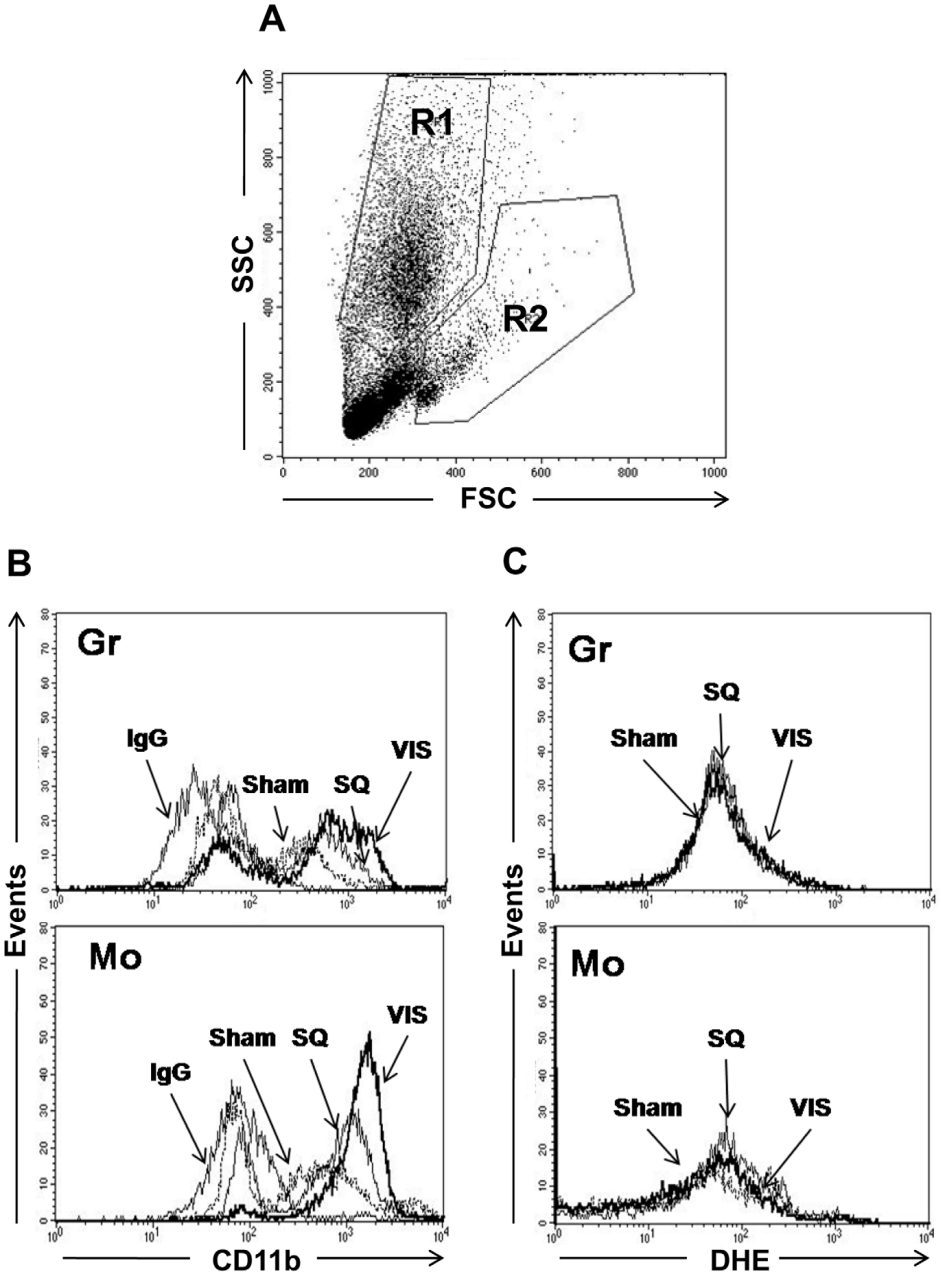
Determination of expression level of cell surface CD11b and oxidative stress in leukocyte subpopulations [granulocytes (Gr), monocyte/macrophage (Mo)]. **A:** Dot plot about gating definition (R1 = granulocyte, R2 = monocyte/macrophage fraction, respectively). **B:** CD11b expression was determined by flow cytometry using the anti-CD11b monoclonal antibody as described in Methods. Each line represents the IgG control, CD11b expression of sham operation group, SQ transplantation group and VIS transplantation group. **C:** Oxidative stress. DHE-associated fluorescence activity was determined in leukocyte subpopulations as described in Methods. Each line represents the DHE intensity of the sham operation group, SQ transplantation group and VIS transplantation group. Data shown are representative of 5 independent experiments.

Since leukocyte integrin expression plays an important role in leukocyte adhesion *in vivo*, we determined the expression of CD11b in granulocytes and monocytes taken from recipient mice after adipose tissue transplantation. As shown in **Figure 4B**, Transplantation of VIS and SQ, to a lesser extent, enhanced CD11b expression in both granulocytes and monocytes. In contrast, DHE-associated oxidative stress was not significantly altered by either type of adipose tissue transplantation in granulocytes or monocytes (**Figure 4C**). To address a causative role of CD11b in leukocyte recruitment induced by adipose tissue transplantation, we injected an anti-CD11b blocking antibody into recipient mouse prior to IVM. The anti-CD11b blocking antibody significantly reduced the number of adherent cells in mice transplanted with SQ and VIS group when compared to control IgG injected group (**Figure S3**).

### Effects of HF on phenotypes of macrophages and monocytes in SVF

**Figure 5:**
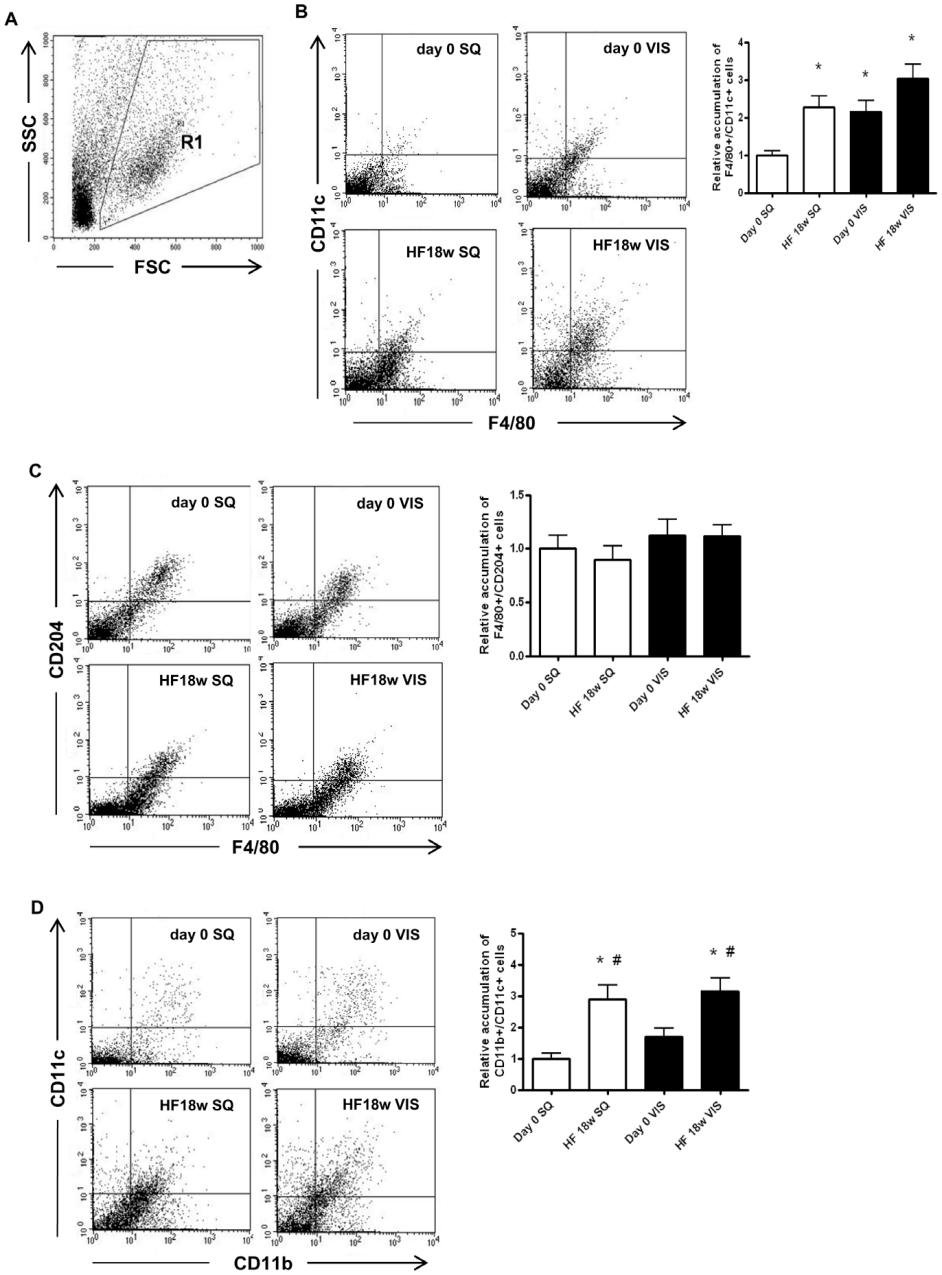
Flow cytometric analysis of monocyte/macrophage subpopulations accumulated in donor mice adipose tissues. **A:** Dot plot about gating definition. The representative dot plots show SQ and VIS adipose tissues from mice at 7 weeks of age (day 0) or fed HF diet for 18 weeks (HF), and the relative amounts of **B:** M1 macrophages (F4/80^+^/CD11c^+^), **C:** M2 macrophages (F4/80^+^/CD204^+^), and **D:** activated monocytes (CD11b^+^/CD11c^+^). Values are shown as the mean ± SEM of 8 mice in each group. *P<0.05 vs. SQ group, ^#^P<0.05 vs. each day 0 group.

The characteristics of the SVF determine the inflammatory phenotype of adipose tissues. Therefore, we isolated SVFs from SQ and VIS, and performed flow cytometric analysis to check the distribution of M1 and M2 macrophages. The number of “classical” M1 macrophages, characterized by F4/80^+^/CD11c^+^, was increased in VIS as compared to SQ. HF 18w significantly enhanced the number of M1 macrophages in both SQ and VIS (**Figure 5B**). In contrast, the number of M2 macrophages, characterized by F4/80^+^/CD204^+^, was not different between the SQ and VIS with or without HF feeding (**Figure 5C**). The total number of infiltrated macrophages (M1+M2) was not so dramatically changed in VIS and SQ with or without HF (**Figure S4**). HF18w also increased the number of activated monocytes (CD11b^+^/CD11c^+^) in the SVF from SQ and VIS (**Figure 5D**).

### Effects of HF on phenotypes of dendritic cells in SVF

**Figure 6:**
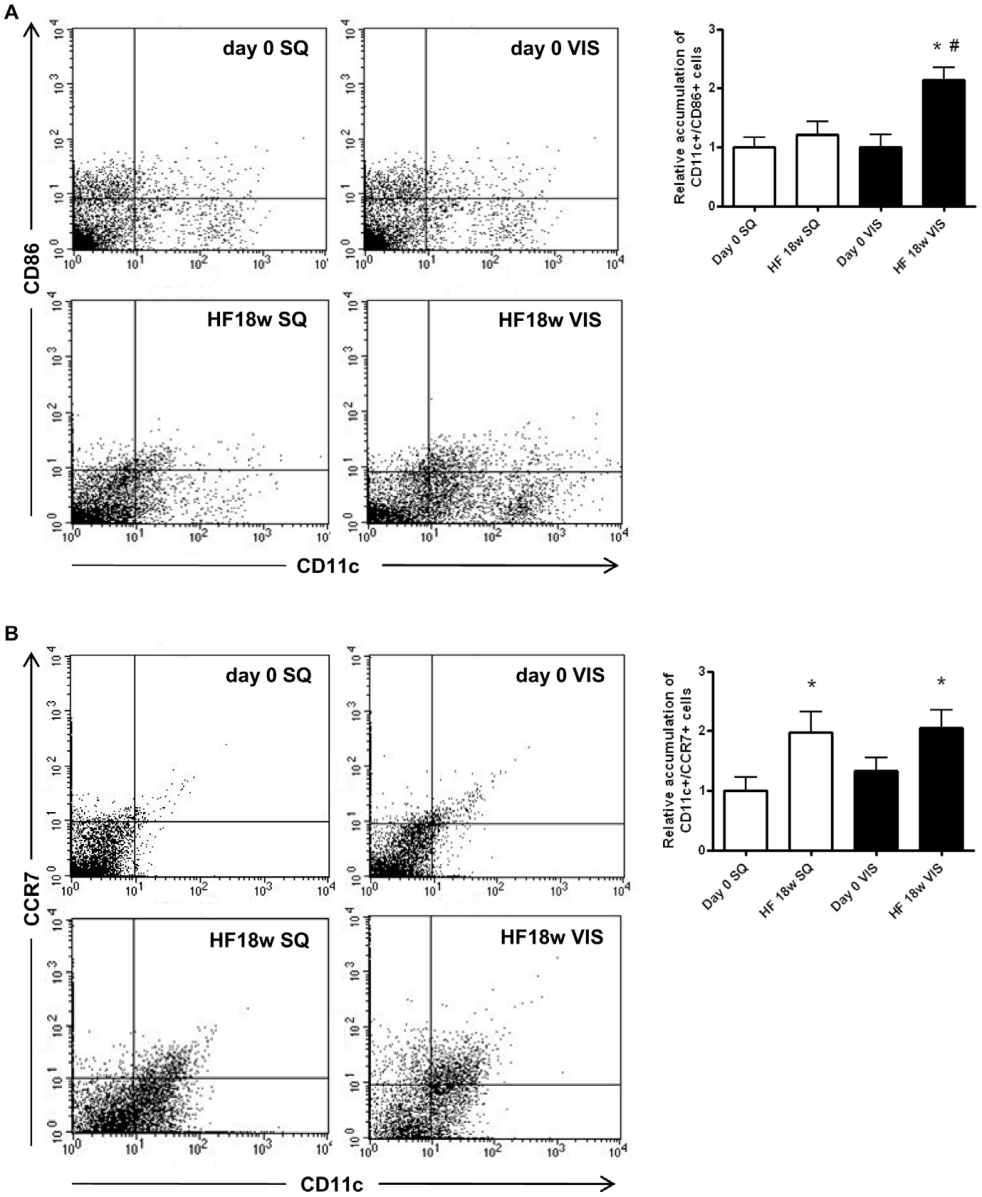
Flow cytometric analysis of subpopulations of mature dendritic cells (DCs) accumulated in donor mouse adipose tissue. The representative dot plots show SQ and VIS adipose tissues from mice at 7 weeks of age (day 0) or fed HF diet for 18 weeks (HF), and relative amounts of **A:** CD11c^+^/CCR7^+^ DCs, **B:** CD11c^+^/CD86^+^ DCs, and **C:** total DCs. Values are shown as the mean ± SEM of 8 mice in each group. *P<0.05 vs. SQ group, ^#^P<0.05 vs. each day 0 group.

The number of DCs, characterized by CD11c^+^/CD86^+^, was increased in VIS adipose tissue but not in SQ adipose tissues of mice fed HF for 18 weeks (**Figure 6A**), while that of those characterized by CD11c^+^/CCR7^+^ was increased in both SQ and VIS adipose tissues of mice fed HF for 18 weeks (**Figure 6B**). When we compared the numbers of macrophages and DCs in the SVF under these conditions, DCs but not macrophages were significantly regulated by HF consumption (**Figure S5**).

## Discussion

Novel findings of this study are: (1) visceral adipose tissue induces more macro-vascular leukocyte adhesion than does subcutaneous adipose tissue; (2) high-fat diet enhances leukocyte adhesion and cytokine expression more prominent in subcutaneous adipose tissue when compared to those in visceral adipose tissue; (3) underlying mechanisms of high-fat diet-induced inflammation in adipose tissue involves recruitment of activated monocytes, M1 macrophages, and DCs.

Leukocyte adhesion is a multi-step complex cascade induced by various factors, including activation of adhesion molecules, production of oxidative stress, and secretion of inflammatory cytokines or chemokines from pro-inflammatory cells, and a crucial mechanism for vascular inflammation and following atherosclerosis. We recently developed a novel IVM system to directly monitor leukocyte recruitment to athero-prone arteries *in vivo*. In the present study, we examined the direct effect of adipose tissue on macro-vascular inflammation using IVM analysis of the femoral artery after adipose tissue transplantation, and noted the possibility of a contribution by adipose tissue in inflammation caused by diet-induced obesity. Our results showed that transplantation of adipose tissue induced leukocyte adhesion to femoral artery with elevation of a pro-inflammatory cytokines and chemokines such as IL-6, MCP-1, and MIP-1β.

Though obesity and vascular inflammation is closely influenced one another and lead to develop atherosclerosis, the direct association between adipose tissue and macro-vascular inflammation such as leukocyte adhesion to femoral artery was not experimentally addressed despite previous studies addressing more chronic effect of adipose tissue in atherosclerosis [28]–[29]. In this regard, our data is the first experimental documentation of pro-inflammatory property of adipose tissue on large blood vessels in vivo. The transplantation of adipose tissues in dorsal area failed to induce comparable leukocyte adhesion to those induced by contra-lateral femoral artery transplantation (data not shown), there may be a distinct and strong pro-inflammatory mechanisms between adipose tissue and vasculature.

As previously reported [30]–[34], high-fat diet induces insulin resistance and various inflammatory conditions in adipose tissues. In our study, SQ was more sensitive to enhance high-fat-triggered leukocyte adhesion when compared to VIC suggesting that the inflammatory status of SQ adipose tissues may contribute to the systemic inflammation under diet-induced obesity.

The accumulation of M1 macrophages and activated monocytes were prominent in VIS as compared to SQ adipose tissues, which were further enhanced by high-fat diet. We also documented an involvement of DCs in adipose tissue inflammation.

Recently, free fatty acid was shown to recruit DCs from bone marrow to adipose tissues via Toll-like receptor 2/4 [35]–[36], while another study reported that adipose tissue is one of the main source of DCs in the body [37]. Therefore, DCs may play a major role in adipose tissue inflammation in close coordination with macrophages in dyslipidemia. Our data are consistent with these reports and confirm the importance of monocytes, macrophages and DCs accumulated in adipose tissues to regulate local inflammation. These findings extend current understanding of relationship between adipose tissue and vasculature in the context of metabolic syndrome and atherosclerosis.

In conclusion, adipose tissue transplantation induced production of inflammatory cytokines and chemokines, resulting in leukocyte adhesion. HF intake enhanced adipose inflammation, including an increase in inflammatory molecules and accumulation of inflammatory cells such as DCs in adipose tissue. Our findings suggest that inflammation caused by adipose tissue directly induces vascular inflammation. Additional studies of the mechanisms that link adipocyte inflammation to vascular inflammation may shed new light on the complex mechanism of atherosclerosis.

## Supporting Information

Figure S1 **Time-dependent analysis of leukocyte adhesive interactions in femoral arteries of mice at 0, 1, 3, 5, and 7 days after SQ or VIS adipose tissue transplantation or sham operation.** Mice that underwent a sham operation without transplantation. Values are shown as the mean ± SEM of 5 mice in each group. *P<0.05 vs. sham group at each time points.
(TIF)

Figure S2 **Body weights of mice before (day 0) and after feeding with normal chow (NC 18w) or high fat diet (HF 18w) for 18 weeks.** *P<0.05 vs. baseline, #P<0.05 vs. NC fed group.
(TIF)

Figure S3 **Effects of anti-CD11b antibody (Ab) in leukocyte adhesive interactions in arteries after adipose transplantation.** The number of adherent cells were quantitated as described in Methods. Values are shown as the mean ± SEM of 4 mice in each group. *P<0.01 vs IgG group.
(TIF)

Figure S4 **Flow cytometric analysis of total macrophages accumulated in donor mice adipose tissue.** The relative amounts of the total numbers of macrophages (M1 macrophages + M2 macrophages) in SQ and VIS adipose tissues from mice at 7 weeks of age (day 0) or fed HF diet for 18 weeks (HF 18w). Values are shown as the mean ± SEM of 8 mice in each group. *P<0.05 vs. SQ group, #P<0.05 vs. day0 SQ.
(TIF)

Figure S5 **Flow cytometric analysis of total DCs accumulated in donor mice adipose tissue.** The relative amounts of the total numbers of DCs (CD86+ DCs + CCR7+ DCs) in SQ and VIS adipose tissues from mice at 7 weeks of age (day 0) or fed HF diet for 18 weeks (HF 18w). Values are shown as the mean ± SEM of 8 mice in each group. *P<0.05 vs. SQ group, #P<0.05 vs. each day 0 group.
(TIF)
