# Supplementary figures and images for: Adipose Inflammation Initiates Recruitment of Leukocytes to Mouse Femoral Artery: Role of Adipo-Vascular Axis in Chronic Inflammation
Source: PLoS One. 2011 May 20;6(5):e19871. doi: 10.1371/journal.pone.0019871 (PMC3098847; doi:10.1371/journal.pone.0019871)

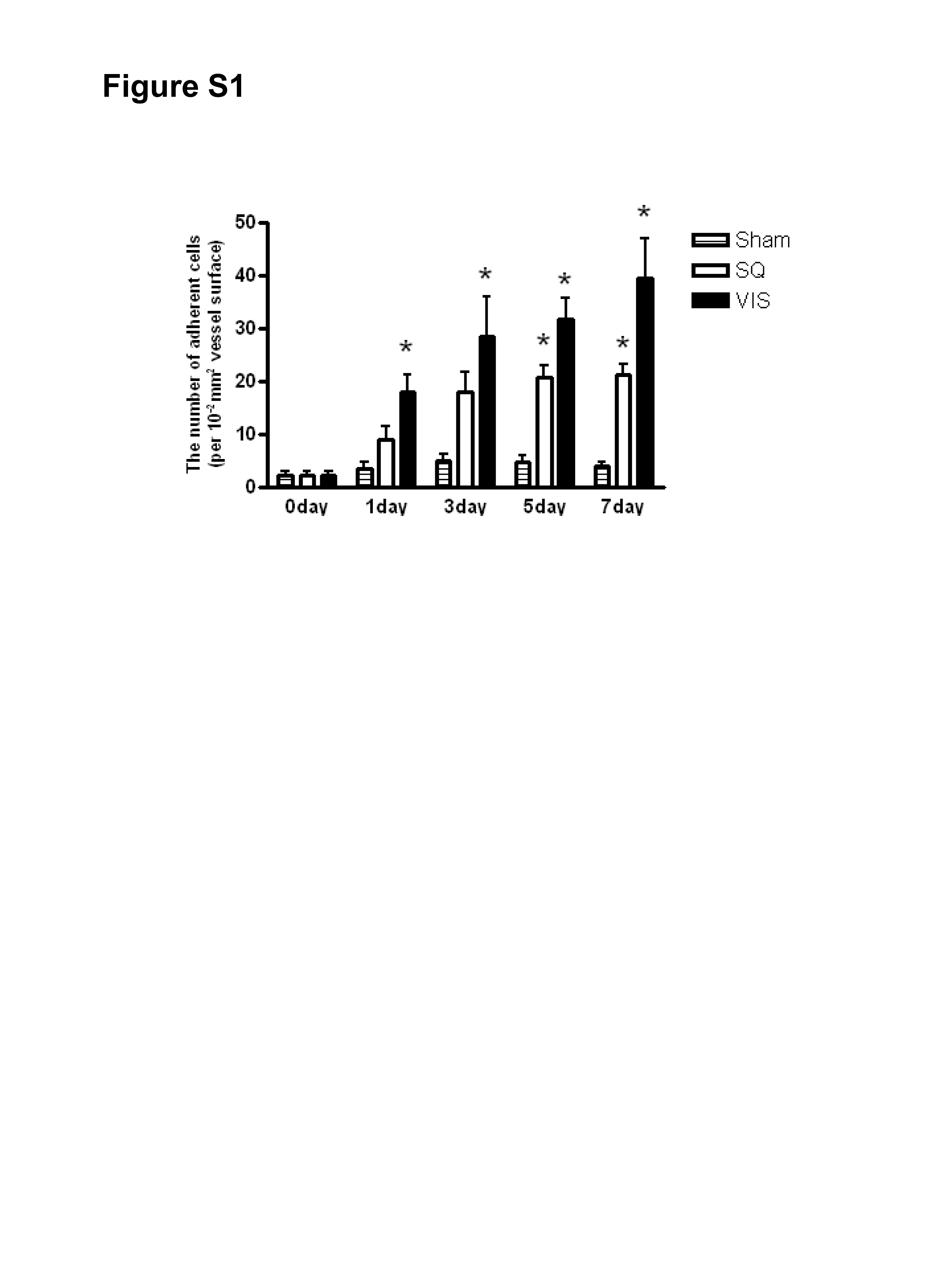

Supplement: Figure S1 — Time-dependent analysis of leukocyte adhesive interactions in femoral arteries of mice at 0, 1, 3, 5, and 7 days after SQ or VIS adipose tissue transplantation or sham operation. Mice that underwent a sham operation without transplantation. Values are shown as the mean ± SEM of 5 mice in each group. *P<0.05 vs. sham group at each time points. (TIF) [file pone.0019871.s001.tif]

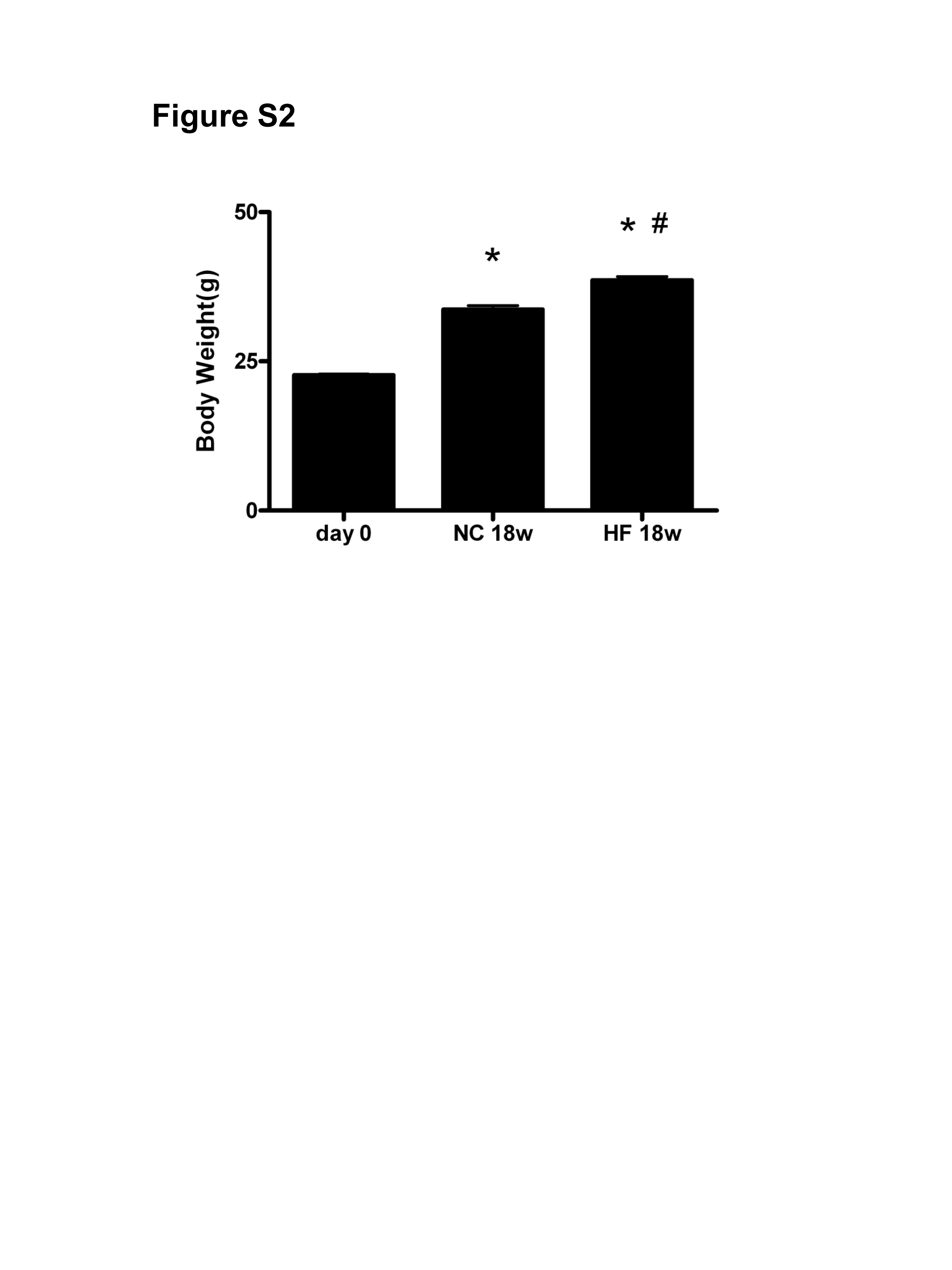

Supplement: Figure S2 — Body weights of mice before (day 0) and after feeding with normal chow (NC 18w) or high fat diet (HF 18w) for 18 weeks. *P<0.05 vs. baseline, #P<0.05 vs. NC fed group. (TIF) [file pone.0019871.s002.tif]

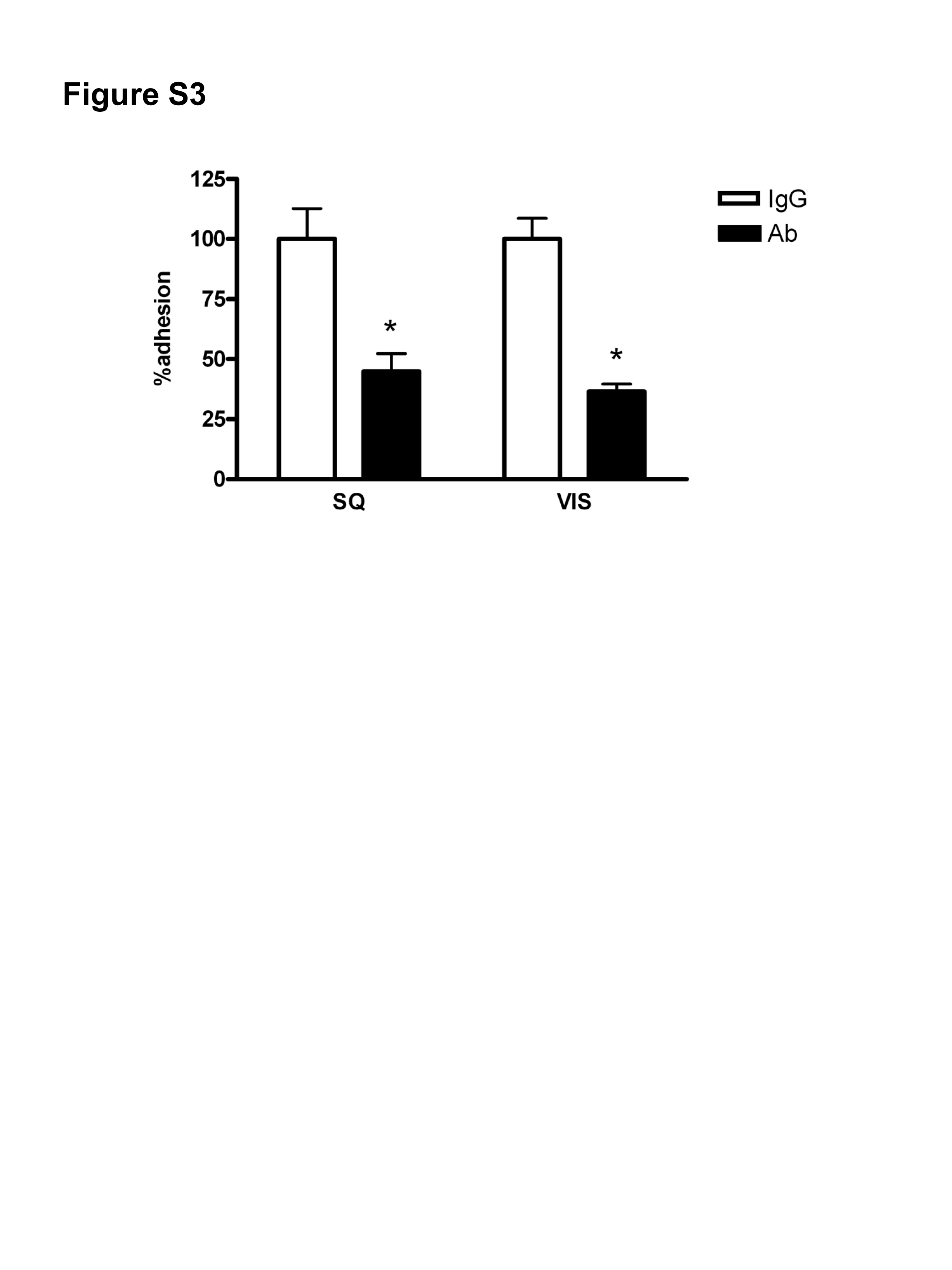

Supplement: Figure S3 — Effects of anti-CD11b antibody (Ab) in leukocyte adhesive interactions in arteries after adipose transplantation. The number of adherent cells were quantitated as described in Methods. Values are shown as the mean ± SEM of 4 mice in each group. *P<0.01 vs IgG group. (TIF) [file pone.0019871.s003.tif]

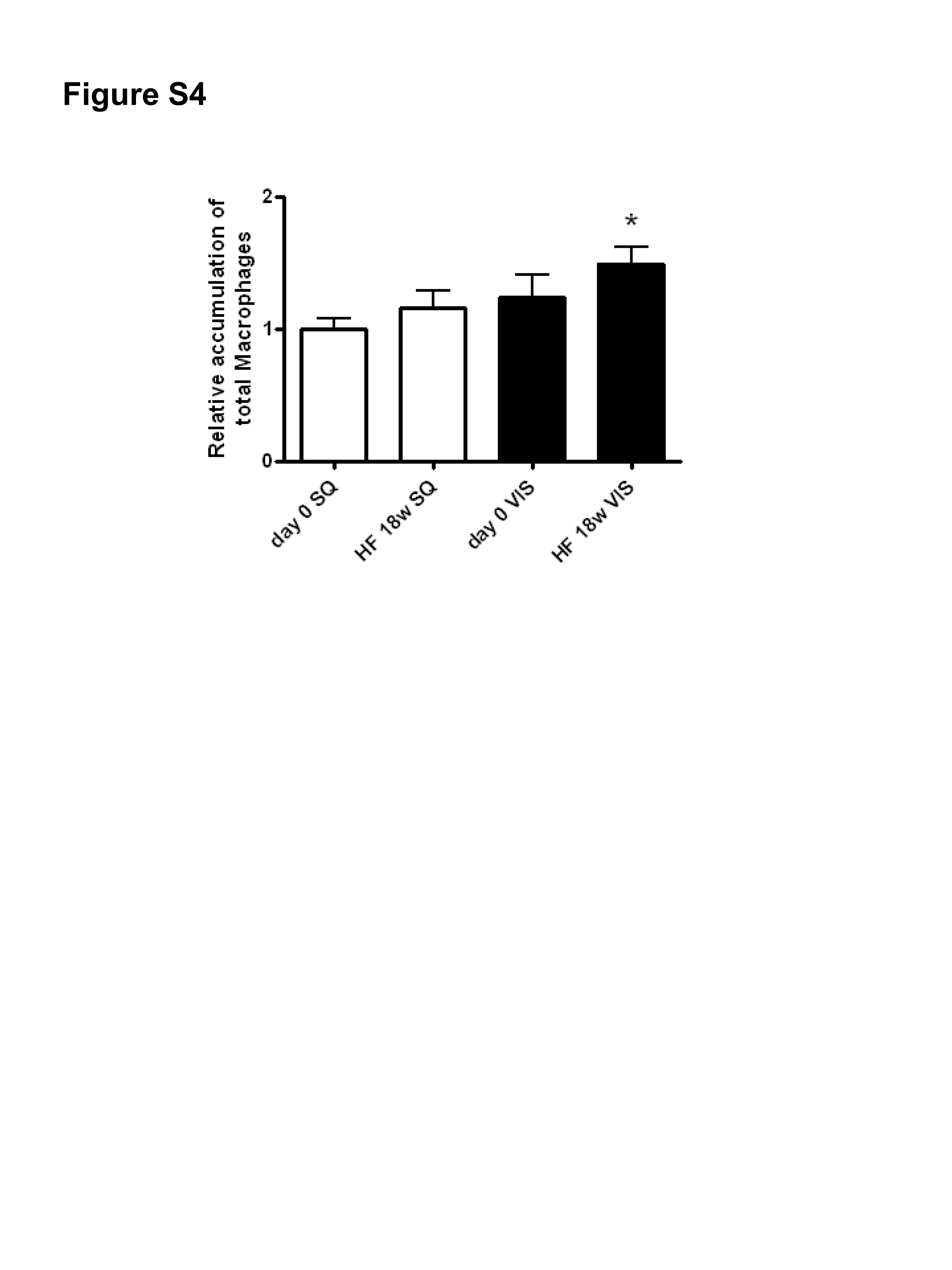

Supplement: Figure S4 — Flow cytometric analysis of total macrophages accumulated in donor mice adipose tissue. The relative amounts of the total numbers of macrophages (M1 macrophages + M2 macrophages) in SQ and VIS adipose tissues from mice at 7 weeks of age (day 0) or fed HF diet for 18 weeks (HF 18w). Values are shown as the mean ± SEM of 8 mice in each group. *P<0.05 vs. SQ group, #P<0.05 vs. day0 SQ. (TIF) [file pone.0019871.s004.tif]

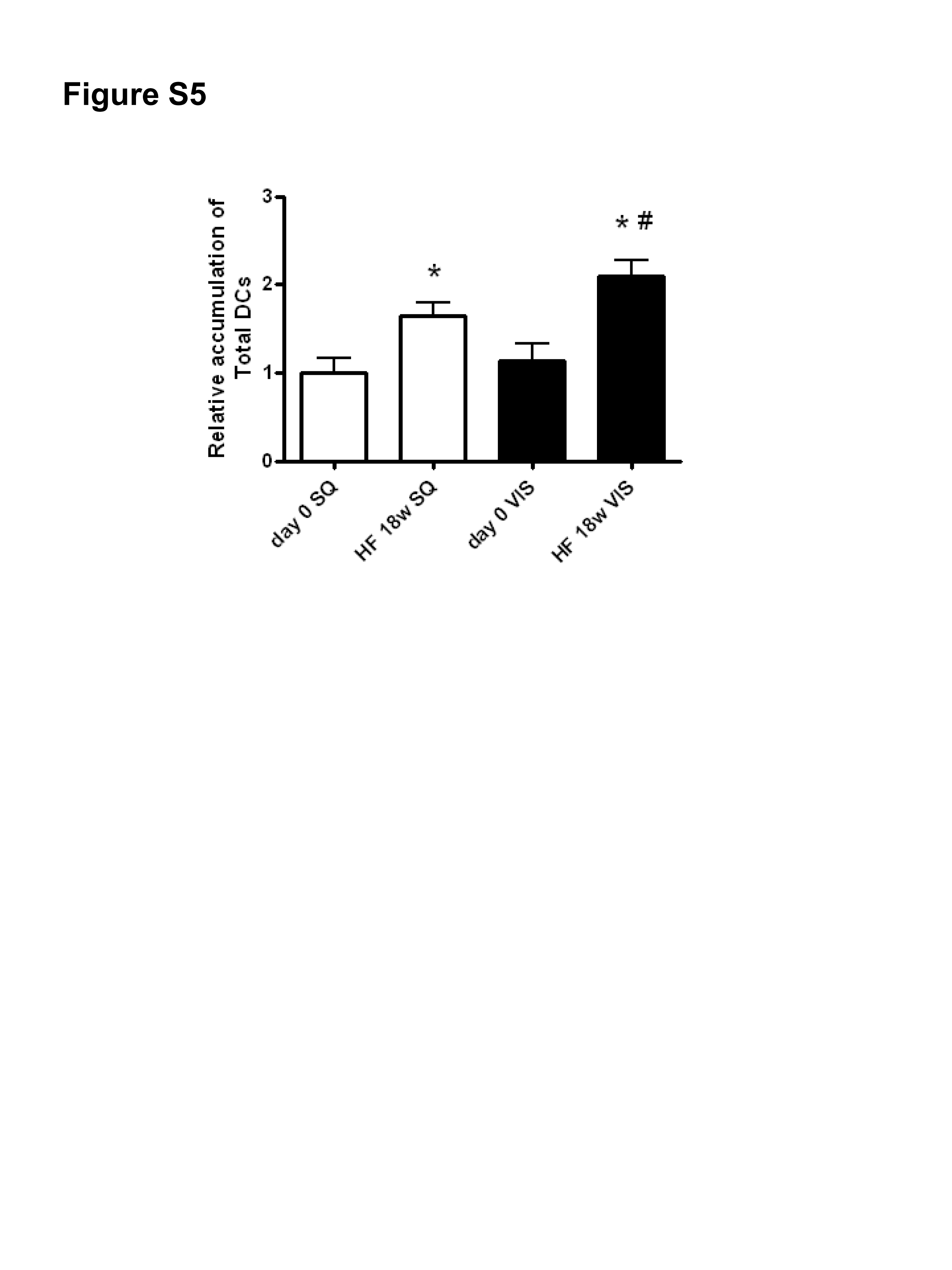

Supplement: Figure S5 — Flow cytometric analysis of total DCs accumulated in donor mice adipose tissue. The relative amounts of the total numbers of DCs (CD86+ DCs + CCR7+ DCs) in SQ and VIS adipose tissues from mice at 7 weeks of age (day 0) or fed HF diet for 18 weeks (HF 18w). Values are shown as the mean ± SEM of 8 mice in each group. *P<0.05 vs. SQ group, #P<0.05 vs. each day 0 group. (TIF) [file pone.0019871.s005.tif]
